# Bacterial outer membrane vesicle-based cancer nanovaccines

**DOI:** 10.20892/j.issn.2095-3941.2022.0452

**Published:** 2022-09-23

**Authors:** Xiaoyu Gao, Qingqing Feng, Jing Wang, Xiao Zhao

**Affiliations:** 1 CAS Key Laboratory for Biomedical Effects of Nanomaterials and Nanosafety & CAS Center for Excellence in Nanoscience, National Center for Nanoscience and Technology of China, Beijing 100190, China; 2 University of Chinese Academy of Sciences, Beijing 100049, China; 3 Center of Drug Evaluation, National Medical Products Administration, Beijing 100022, China; 4 IGDB-NCNST Joint Research Center, Institute of Genetics and Developmental Biology, Chinese Academy of Sciences, Beijing 100101, China

**Keywords:** Cancer, cancer vaccines, outer membrane vesicles, nanocarriers, tumor antigen

## Abstract

Tumor vaccines, a type of personalized tumor immunotherapy, have developed rapidly in recent decades. These vaccines evoke tumor antigen-specific T cells to achieve immune recognition and killing of tumor cells. Because the immunogenicity of tumor antigens alone is insufficient, immune adjuvants and nanocarriers are often required to enhance anti-tumor immune responses. At present, vaccine carrier development often integrates nanocarriers and immune adjuvants. Among them, outer membrane vesicles (OMVs) are receiving increasing attention as a delivery platform for tumor vaccines. OMVs are natural nanovesicles derived from Gram-negative bacteria, which have adjuvant function because they contain pathogen associated molecular patterns. Importantly, OMVs can be functionally modified by genetic engineering of bacteria, thus laying a foundation for applications as a delivery platform for tumor nanovaccines. This review summarizes 5 aspects of recent progress in, and future development of, OMV-based tumor nanovaccines: strain selection, heterogeneity, tumor antigen loading, immunogenicity and safety, and mass production of OMVs.

## Introduction

Cancer is a major global public health threat^1^. As technology advances, cancer therapy has undergone a paradigm shift^2^. In traditional therapies, including surgery, radiotherapy, and chemotherapy, all patients are treated in a similar mode, and the treatments not only kill tumor cells but also harm normal cells^3^. With advances in basic research in cancer, precision therapy, represented by molecular targeted therapy and immunotherapy, has gradually become a focus of cancer therapy. Molecular targeted therapy, such as monoclonal antibodies or inhibitors against oncogenes, and immunotherapy, such as adoptive cell therapy and immune checkpoint blocking therapy, have shown strong anti-tumor activity in a variety of tumor types^4–6^. Precision therapy can target a group of patients with a model of treatment, but potential limitations remain, such as low clinical response rates and off-target lethality^7^. The future development direction of tumor therapy will involve formulating individual treatment models for each patient^8^. Cancer cells are the malignant products of the accumulation of gene mutations, and each patient has a unique genetic mutation spectrum. Developing specific treatments against each patient’s mutated gene spectrum would allow truly personalized cancer therapy to be achieved.

Although the development of highly safe and effective personalized cancer therapies requires substantial resources and time^9^, the establishment of tumor vaccine platforms can shorten the development trajectory^10^. In recent years, outer membrane vesicles (OMVs) have gradually been applied to personalized tumor vaccine platforms. The cargoes originating from the donor bacteria, particularly pathogen associated molecular patterns (PAMPs), enable OMVs to activate a variety of Toll-like receptor (TLR) signaling pathways^11^. This potent immunogenicity distinguishes OMVs as promising delivery vectors for tumor vaccines. In this review, we summarize tumor vaccines and tumor nanovaccines, and introduce OMVs and their applications in tumor vaccines. Specifically, we highlight 5 aspects of the current progress in, and future development of, OMV-based tumor nanovaccines: strain selection, heterogeneity, tumor antigen loading, immunogenicity and safety, and mass production of OMVs.

## Tumor vaccines

With the rapid technological advances in next generation sequencing, genomics, bioinformatics and other fields, the mutated peptides generated from gene mutations in cancer cells can be identified, some of which have the potential to activate immune responses as so-called tumor antigens^12,13^. Tumor antigens are classified into tumor-associated antigens and tumor-specific antigens. Tumor-associated antigens are proteins that are highly expressed by tumor cells, whereas tumor-specific antigens are gene mutation-generated neoantigens^14^. Tumor-specific antigens are usually referred to as tumor antigens. Because these tumor antigens are specific to tumor cells, treatments that target them do not harm normal cells^12^.

An emerging tumor treatment modality, therapeutic tumor vaccines, has been developed against specific tumor antigens. Through presenting tumor antigens to the immune system, therapeutic tumor vaccines activate tumor antigen-specific T cells that specifically recognize and kill tumor cells^15,16^. More than 200 clinical trials of cancer vaccines have been performed worldwide, and studies on tumor vaccines are increasingly being performed^17^. In April 2010, Provenge/sipuleucel-T, the first therapeutic tumor vaccine approved by the U.S. Food and Drug Administration, was applied to treat advanced prostate cancer^18^. In 2017, 2 groups from the Dana-Farber Cancer Institute in the United States and Johannes Gutenberg University Mainz in Germany made major breakthroughs in the field of tumor vaccines. After predicting possible tumor antigens through bioinformatic technologies, the researchers synthesized 2 tumor vaccines based on peptides or mRNA, which yielded encouraging results in patients with advanced melanoma^19–21^. Therapeutic tumor vaccines are personalized tumor therapies that target each patient’s unique genetic alterations, and not only activate tumor antigen-specific T cells but also amplify existing anti-tumor immunity.

## Tumor nanovaccines

When tumor antigens are used alone, the immunogenicity of tumor vaccines is low and consequently is insufficient to activate effective anti-tumor immune responses. Efficient tumor vaccines often require immune adjuvants and nanocarriers to enhance the immunogenicity of tumor antigens^17^. Nanoparticle-based vaccine delivery systems, also called nanovaccines, are nanomaterials (20–100 nm) that target the human immune system and activate the host’s immune responses against diseases. Antigens and adjuvants are conjugated to the surface or core of nanoparticles with various materials and manufacturing conditions^22^. Immune adjuvants are usually stimulants of innate immunity that act on antigen presenting cells (APCs), thus providing the necessary co-stimulatory signals for successful antigen presentation. Examples include TLR agonists, dendritic cell (DC) targeting monoclonal antibodies, saponin adjuvants, granulocyte macrophage colony-stimulating factor, stimulator of interferon genes (STING) ligands, aluminum hydroxide, or lipopolysaccharide (LPS)^23^. Nanocarriers use the natural uptake pathways of immune cells to enhance the uptake and processing efficiency of neoantigens by APCs^24^. Several materials have been used to develop nanocarriers for tumor vaccine delivery, such as lipids^25^, polymers^26^, synthetic high-density lipoproteins^27^, and DNA origami^28^. To ensure that innate immune activation and antigen delivery occur in the same APCs, current tumor nanovaccines are designed to use nanocarriers to co-deliver tumor antigens and immune adjuvants.

The co-delivery design of nanovaccines requires complex synthesis processes and the addition of adjuvants. To avoid additional components and preparation steps, tumor vaccine carriers often integrate nanocarriers and immune adjuvants. Some promising nanocarriers with intrinsic immune adjuvant properties, such as polymeric and lipid nanoparticles that activate the STING pathway, have been developed^29,30^. These nanovaccines do not require the addition of exogenous adjuvants, and they effectively inhibit tumor growth in a variety of tumor models. However, these nanomaterials with unique adjuvant properties require complex screening and synthesis.

## OMVs

In recent years, biomimetic nanomaterials, particularly natural biofilms, have attracted increasing attention from researchers^31^. Because biomimetic nanomaterials have similar surface proteins and complete phospholipid bilayers to natural biofilm, they have special functions such as ligand recognition, biological targeting, and long circulation times. Among them, OMVs, natural spherical nanovesicles, are from Gram-negative bacteria and have a diameter of 30–200 nm^32^. OMVs can be functionally modified by genetic engineering, are rich in PAMPs, and can activate multiple TLR signaling pathways^33^. In 1967, Chatterjee and Das^34^ discovered OMVs while studying the cell wall structure of *Vibrio cholerae in vitro*. Since then, OMVs have been observed in an increasing number of Gram-negative bacteria^35^. At present, no definite conclusions have been drawn regarding the biogenesis of OMVs. However, several biogenesis pathways have been reported^36^, such as (1) disruption of peptidoglycan-lipoprotein crosslinks^37^; (2) accumulation of envelope components^38^; (3) enrichment of specific LPS in some areas^39^; (4) insertion of the pseudomonas quinolone signal^40^; and (5) downregulation of the VacJ/Yrb ABC transporter^41^. Many studies have shown that the biogenesis of OMVs can aid in bacterial defense against antibiotics such as gentamicin^42^ through antibiotic dilution, increase the survival rate of bacteria in other harsh environments, deliver virulence factors to the host, increase bacterial immune evasion after host infection^32^, and transfer genes, such as those conferring antibiotic resistance, to help bacteria better adapt to the environment^43^.

**Figure 1:**
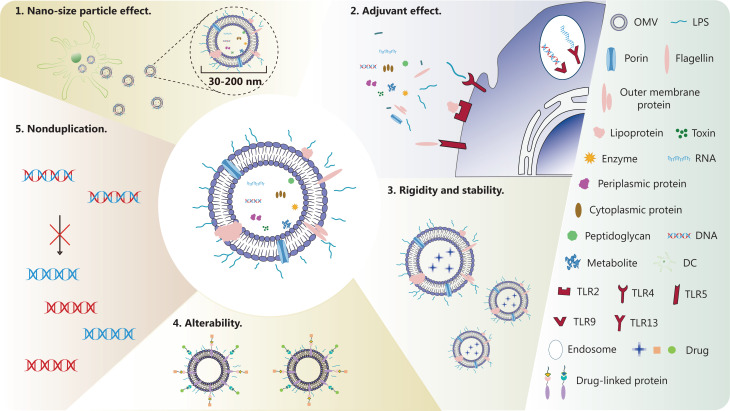
Composition and advantages of OMVs. OMVs are composed of LPS, porin, flagellin, outer membrane protein, lipoprotein, toxin, enzyme, periplasmic protein, cytoplasmic protein, peptidoglycan, metabolites, DNA and RNA. Compared with traditional tumor vaccine nanocarriers, OMVs have the following clear advantages: 1. Owing to the nano-sized particle effect and their exogenous nature, OMVs can be quickly recognized and taken up by DCs. 2. OMVs are rich in many PAMPs and can activate multiple innate immune signaling pathways, thereby exerting a natural adjuvant effect. 3. OMVs are stable and rigid, and can decrease the degradation of loading antigens. 4. OMVs can be functionally enhanced and modified by genetic engineering of bacteria. 5. Unlike weakened bacteria, OMVs cannot replicate and therefore are more secure.

OMVs are nonreplicating nano-sized vesicles composed of lipids, outer membrane proteins, periplasmic proteins, DNA and RNA. OMVs are rich in bacterial PAMPs, such as LPS, peptidoglycan, and flagellin^44^. LPS is an endotoxin and a highly potent PAMP. After binding the pattern recognition receptor (PRR) TLR4^45^, LPS simultaneously activates the downstream signaling pathways of MyD88 and TRIF, thus leading to the release of inflammatory factors and activation of the innate immune response^46^. In addition, bacterial lipoprotein stimulates TLR2 responses; flagellin stimulates TLR5 responses; unmethylated bacterial CpG DNA stimulates TLR9 responses; and bacterial ribosomal RNA stimulates TLR13 responses^11^. In summary, OMVs activate different TLRs, thus inducing the innate immune response. Consequently, OMVs have gradually become highly promising candidate vaccine nanocarriers. To obtain high-quality OMVs, many techniques have been developed to isolate and purify OMVs, such as ultracentrifugation, ultrafiltration, precipitation, size-exclusion chromatography, affinity isolation, and density gradient centrifugation^36^. Compared with traditional tumor vaccine nanocarriers, OMVs have several clear advantages: (1) owing to the nano-sized particle effect^32^ and their exogenous nature, OMVs can be quickly recognized and taken up by DCs; (2) OMVs are rich in many PAMPs and can activate multiple innate immune signaling pathways, thus resulting in a natural adjuvant effect^35^; (3) OMVs are stable and rigid, and can decrease the degradation of loading antigens^47^; (4) OMVs can be functionally enhanced and modified through genetic engineering of bacteria^47^; (5) in contrast to weakened bacteria, OMVs cannot duplicate and therefore are more secure^36^ (**Figure 1**).

## Current progress in OMV-based tumor nanovaccines

**Figure 2:**
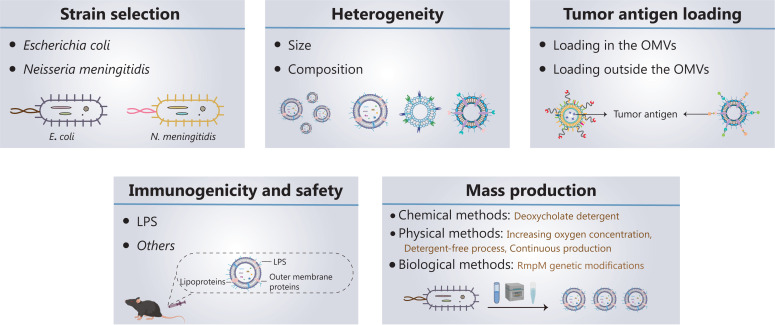
Current progress in, and future development of, OMV-based tumor nanovaccines. Five key aspects must be developed and investigated for OMVs to be used as tumor vaccine vectors: (1) strain selection; (2) heterogeneity of OMVs; (3) tumor antigen loading; (4) immunogenicity and safety of OMVs; and (5) mass production of OMVs.

Five key aspects must be developed and investigated when OMVs are used as tumor vaccine vectors (**Figure 2**): (1) strain selection; (2) heterogeneity of OMVs; (3) tumor antigen loading; (4) immunogenicity and safety of OMVs; and (5) mass production of OMVs.

### Strain selection

OMVs derived from *Escherichia coli* (*E. coli*), *Klebsiella pneumoniae*, *Salmonella typhimurium*, and attenuated *Salmonella* have been used as drug delivery vehicles or directly in tumor therapy^48–50^. The OMVs currently used in tumor vaccines are all derived from *E. coli*, because of the ease of genetic engineering of *E. coli*^51,52^. However, *E. coli*-derived OMVs present 2 main problems: (1) *E. coli* exists in the human body, and a potential exists for immune tolerance to *E. coli* OMVs, and (2) the strong immune responses against *E. coli* OMVs may affect the normal flora of the intestinal tract. The proposed *Neisseria meningitidis* (*N. meningitidis*) OMV-based vaccines have been approved and successfully used to prevent outbreaks of *N. meningitidis*^53–55^. Therefore, OMVs from *N. meningitidis* are highly immunogenic and have been approved as safe; consequently, they may serve as ideal nanocarriers for tumor vaccines. However, further research is needed to confirm this possibility.

### Heterogeneity of OMVs

OMVs vary widely in size and composition, depending on their endogenous and exogenous sources. Endogenous factors mainly include the selection of bacterial strains and biogenesis pathways; exogenous factors mainly include the growth stage, growth environment, and extraction method. OMV composition varies among strains. For example, in *E. coli*-derived OMVs^56^ phosphatidylethanolamine is the main phospholipid, whereas in *Helicobacter pylori* (*H. pylori*)-derived OMVs, the content of phosphatidylethanolamine is relatively low, and diphosphatidylglycerol is the main phospholipid^57^. A total of 141 protein components are present in *E. coli*-derived OMVs^58^, whereas 57 protein components are present in *N. meningitidis*-derived OMVs^59^. Hong and colleagues^60^ have analyzed the OMV proteomes from 2 *E. coli* strains, compared with bacterial cultures grown in iron-restricted and iron-supplemented conditions. The authors observed differences in the composition of OMVs according to *E. coli* strain and growth environment^60^. Zavan et al.^61^ have shown that the composition of *H. pylori*-derived OMVs varies among growth stages.

OMVs are heterogeneous, showing differences in size, protein composition, and content. These differences may cause OMVs to target different host cells and elicit different biological effects. A recent study has indicated that bacterial growth stage affects the ability of OMVs to induce the production of IL-8 by human epithelial cells^61^. Unfortunately, the production conditions and preparation methods of OMVs lack standardization; consequently, the composition and size of each batch of OMVs vary greatly and limit the clinical applications of OMVs. Transmission electron microscopy, nanoparticle tracking analysis, and mass spectrometry are often used to observe the morphology, detect the particle size and zeta potential, and analyze the composition of OMVs, respectively^60^. Ideal OMVs should have a low polydispersity index (PDI, < 0.2). Another quality control measure in the production of future OMV-based products may be the presence of characteristic proteins in OMVs, such as outer membrane protein A/C/F, which can be used as a fingerprint for OMV identification.

### Tumor antigen loading

**Figure 3:**
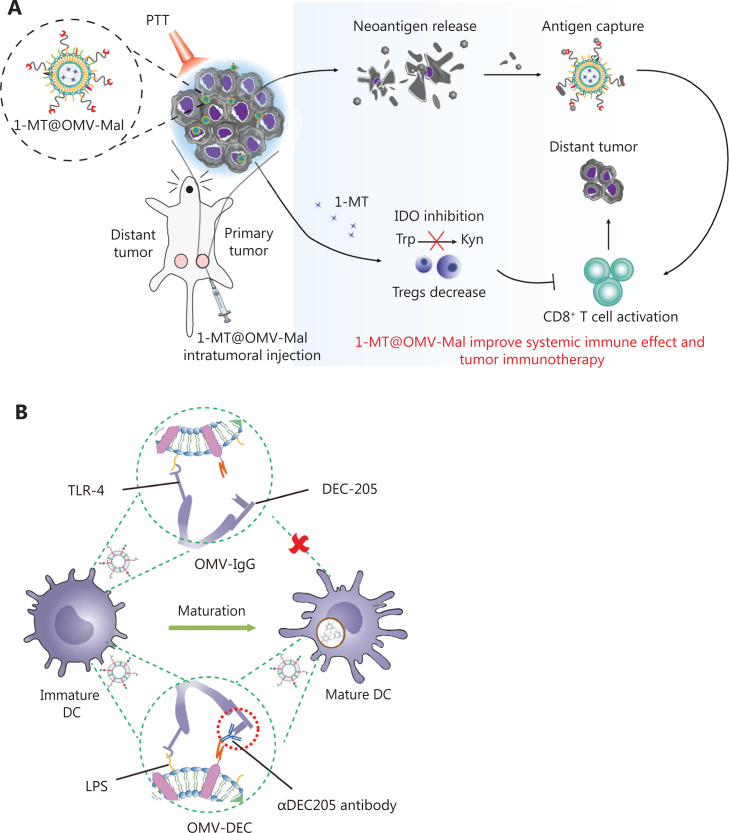

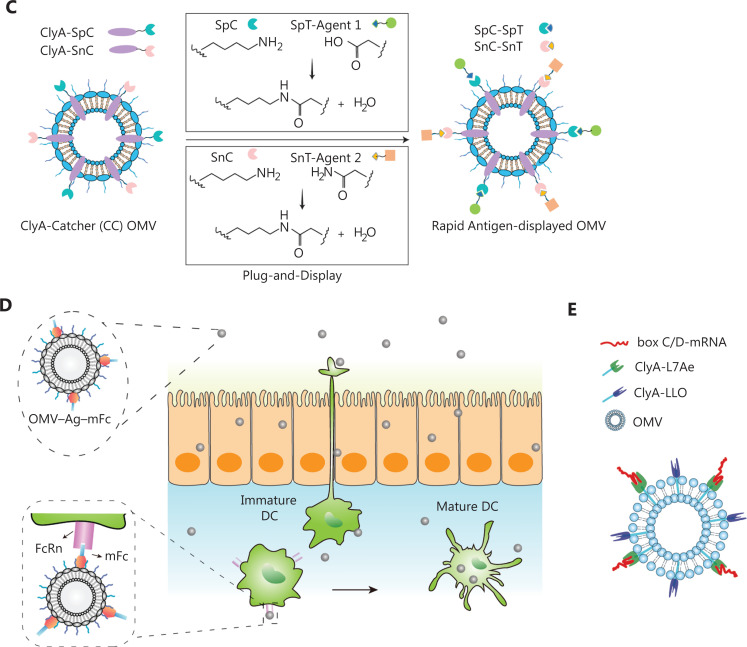
Application of OMVs in tumor vaccines. (A) OMVs facilitate immune-mediated tumor clearance after photothermal therapy through orchestrating antigen capture and immune modulation. Reproduced with permission from reference 47. Copyright 2022, Small. (B) OMVs with the DC-targeting αDEC205 antibody can overcome maturation-induced uptake obstruction. Reproduced with permission from reference 59. Copyright 2022, Fundamental Research. (C) A versatile OMV-based vaccine platform can rapidly and simultaneously display multiple tumor antigens, and consequently elicit synergistic anti-tumor immune responses for personalized tumor vaccines. Reproduced with permission from reference 60. Copyright 2021, Nature Communications. (D) Engineered OMVs release tumor antigens in the intestines after oral administration, thereby activating strong anti-tumor immune responses and immune memory effects. Reproduced with permission from reference 48. Copyright 2022, Nature Biomedical Engineering. (E) OMVs can be used as an mRNA delivery platform to activate strong anti-tumor immune responses. Reproduced with permission from reference 61. Copyright 2022, Advanced Materials.

In recent years, OMVs has gradually been applied to deliver tumor antigens. Current strategies for surface modification or drug loading of OMVs include physical mixing^48^, electroporation^62^, and protein fusion^63^. For example, Li et al.^51^ have designed a promising *in situ* vaccine based on OMVs. The authors used maleimide (Mal) groups to modify the surfaces of OMV-Mal through a reaction between Mal-PEG4-NHS and amine groups on membrane proteins of OMVs. Then indoleamine 2, 3-dioxygenase inhibitor 1-methyl-tryptophan (1-MT) was loaded into the OMV-Mal by electroporation, thus forming 1-MT@OMV-Mal. The vaccines have been found to facilitate immune-mediated tumor clearance after photothermal therapy through orchestrating antigen capture and immune modulation (**Figure 3A**). However, owing to the heterogeneity caused by new and frequent mutations, and the genetic instability of tumors, the development of personalized therapeutic tumor vaccines has gradually become a topic of interest in tumor therapy^64^. The antigen loading of personalized vaccines is based on a “plug and play” technology, in which the OMV membrane protein ClyA is fused to catcher proteins, and the tag-labeled antigens are displayed on the surfaces of OMVs *via* tag/catcher protein pairs^65,66^. Liang et al.^65^ have decorated OMVs with the DC-targeting αDEC205 antibody (OMV-DEC), thus endowing the nanovaccine with an uptake mechanism that is not restricted to maturation *via* antibody modification, thereby overcoming the phenomenon of maturation-induced uptake obstruction. In addition, the authors have used molecular glue technology to build a “plug-and-display” function in an OMV-based nanocarrier, thus achieving rapid display of tumor antigens and developing a personalized tumor vaccine (**Figure 3B**). Cheng et al.^66^ have described a versatile OMV-based vaccine platform to elicit specific anti-tumor immune responses *via* specifically presenting antigens on OMV surfaces. The platform can rapidly and simultaneously display multiple tumor antigens to elicit synergistic anti-tumor immune responses for personalized tumor vaccines (**Figure 3C**). Most current OMV-based vaccines for cancer are injectable vaccines. A major challenge in the field of OMV-based vaccines is changing the route of administration of OMV-based vaccines and increasing patient compliance. Recently, Yue et al.^52^ have fused the tumor antigen and Fc fragment of mouse IgG to the C-terminus of the surface protein ClyA, thus allowing this genetically engineered bacterial robot to secrete OMVs with tumor antigens under the induction of arabinose. The bacterial robot can overcome the harsh digestive tract environment and reach the intestines after oral administration. Oral arabinose induces the bacterial robot to produce OMVs carrying tumor antigens *in situ* in the intestines, thereby activating strong anti-tumor immune responses and immune memory effects (**Figure 3D**). To expand the application of tumor vaccine types, Li et al.^67^ have modified RNA binding protein on the surfaces of OMVs through fusion protein expression, and used box C/D sequence-labeled mRNA antigens to adsorb antigens on the surfaces of OMVs. This is the first time that OMVs were successfully been used as an mRNA delivery platform. This general mRNA tumor vaccine design has promising application prospects (**Figure 3E**). Unfortunately, OMV-based tumor vaccines are still in preclinical stage, and their anti-tumor effects have been detected only at the animal level. Translating OMV-based tumor vaccines into clinical applications remains a major challenge.

### Immunogenicity and safety of OMVs

LPS is the main potential toxic component in OMVs, so it is immunogenic and immunoreactive ^36^. Currently, the clinically approved OMV-based *N. meningitidis* vaccines use deoxycholate detergent to decrease LPS and ensure safety. However, this detergent method causes the loss of important lipoproteins on the surfaces of OMVs, thus decreasing immunogenicity^68^. Another strategy to obtain OMVs with low LPS toxicity is genetic engineering of LPS to reduce the acyl chain or phosphate group (such as msbA, msbB, Imp, lpxL1, lpxM, pagL and so on)^69^. Bos and colleagues^70^ have found that OMVs extracted by removal of the Imp gene have similar LPS levels to OMVs extracted with the detergent method, but without loss of lipoproteins on the surfaces of OMVs. Moreover, mutating hexa-acylated LPS to penta-acylated LPS decreases the toxicity of LPS and improves the safety of OMVs^71^. Zariri et al.^46^ have found that inactivation of the lpxL1 gene or expression of the pagL gene can form penta-acylated LPS. The detergent method inhibits both TLR4 and TLR2 activation by OMVs, whereas the genetic engineering of LPS decreases only TLR4 activation. In addition, the encapsulation method can decrease inflammatory responses due to systemic exposure of OMVs after intravenous injection. Qing et al.^72^ have encapsulated OMVs with a pH-sensitive shell of calcium phosphate, thus decreasing toxic adverse effects and achieving tumor-targeted release. LPS-deficient OMVs exert lower immunogenicity than OMVs with normal LPS levels. Consequently, a new challenge involves identifying the ideal balance between low toxicity and high immunogenicity.

In addition, other components of OMVs, such as outer membrane proteins and lipoproteins, can induce systemic inflammatory responses^47^. To improve the safety of OMVs, Zheng et al.^73^ have designed a synthetic adjuvant carrier that morphologically mimics bacteria and comprises an optimal combination of components derived from bacterial cell walls, flagella, and nucleoids. The bacterium-mimicking vectors (BMVs) cooperatively trigger multiple PRR signaling pathways, and display anti-tumor therapeutic and prophylactic effects superior to those of either the reported synthetic or bacterium-derived adjuvant. Importantly, the synthetic BMVs with detoxified and controllable composition exhibit diminished toxicity. Unfortunately, the main hurdle in synthetic BMVs is choosing the optimal combination of PAMPs to decrease toxic adverse effects and further increase the immunogenicity of the adjuvants, in fine-tuning the desired immune responses to achieve the best anti-tumor effects.

### Mass production of OMVs

During production, the scalability of OMVs is critical to ensure economic viability. The extraction of OMVs relies primarily on ultracentrifugation, ultrafiltration, protein precipitation, and size exclusion chromatography^69^. However, these methods are limited to small-scale production, and their yields are low. Currently marketed OMV-based vaccines exhibit potent endotoxicity, such as that from LPS. To remove most LPS and increase the safety of OMVs, the production process requires deoxycholate detergent extraction. The resulting OMVs tend to aggregate, and the deoxycholate cannot be completely removed during the purification process, thus posing safety hazards. In addition, the production process of OMVs is complicated, and toxic chemicals such as phenol are used, thereby limiting the large-scale production of OMVs^74^.

To decrease the cost of producing OMVs, researchers have increased the yield of OMVs and improved the production process. The rmpM gene maintains bacterial cell wall stability through connecting the outer membrane protein and peptidoglycan layer^75^. Waterbeemd et al.^74^ have found that knockout of the rmpM gene increases OMV production. Gerritzen et al.^76^ have shown that the productivity of OMVs is improved by slowly increasing oxygen concentrations during bacterial culture. Because oxygen concentration is a controllable process parameter in bacterial culture, it can be used as a convenient process parameter to induce OMV release. In 2013, Waterbeemd et al.^77^ developed an improved detergent-free process to produce OMVs. The use of sterile equipment replaced the undesirable steps of ultracentrifugation, phenol inactivation, and the use of preservatives. The process is more consistent than other methods, by providing better stability and higher yield. In 2019, Gerritzen et al.^78^ developed a method for continuous production. Compared with batch production, the volumetric productivity of continuous culture reached 4.0 × 10^14^ OMVs/L/d. However, these improved production methods have been used to produce OMV-based vaccines against *N. meningitidis*, and their efficacy and immunogenicity as tumor vaccine vectors requires further study.

## Concluding remarks and future perspectives

As described in the above sections, OMVs have promise in applications in tumor vaccines. OMVs can integrate the carriers and adjuvants of tumor vaccines, activate multiple natural immune signaling pathways, and prevent or eliminate tumors effectively. However, OMVs will require extensive further development before they can be used as tumor vaccine adjuvants and carriers in clinical settings, such as standardizing the operating guidelines of OMVs, decreasing the production cost of OMVs, increasing the safety of OMVs, and using OMVs for personalized treatment. At present, only *E. coli* is used to prepare OMVs as a tumor vaccine platform. However, identifying other engineered strains to prepare OMVs might enable a better balance between the safety and immunogenicity of OMVs. In addition, no clear conclusions have been drawn regarding the biogenesis of OMVs, and the biological signals and pathways activated *in vivo*. Future studies are expected to begin to address these fundamental questions. Furthermore, a polyethylene glycol (PEG) layer might be modified on OMV surfaces to make OMVs less toxic when they circulate in the body, thus achieving better therapeutic effects. Overall, OMVs warrant further research and greater attention to complement the carriers and adjuvants of tumor vaccines and develop safer, more effective tumor vaccines.
